# The Institutional Library

**Published:** 1921-03-12

**Authors:** 


### THE INSTITUTIONAL LIBRARY.

**Industrial Colonies for the Consumptive.** By Sir German Woodhead, M.D., and P. Varrier-Jones, M.R.C.S., L.R.C.P., with Preface by Sir Clifford Allbutt. Pp. 151 + ix. (University Press, Cambridge. 1920.)

Industrial colonies for consumptives are helpful things, and in founding a thriving one in Cambridgeshire the authors have done well. But it is questionable whether this book of theirs is quite so good a performance. As Dr. Stanley Tinker has easily demonstrated, the task of providing suitable life and trade conditions for the huge numbers of consumptives in this country is obviously out of all proportion to an undertaking which in five years, albeit considerably helped by military and naval pensions, has only treated eighty-eight patients. The title, also, is a little ambitious; there is small reference to foreign literature or to previous work, while generalities make up the bulk of the book. However, there is only one serious error in it (although we think a fuller knowledge of that morbid individual, J. A. Symonds, would have shown the authors that his nervous troubles ante-dated his phthisis, and were, in fact, the result of a congenitally abnormal temperament). This error is the energetic denial of the eugenic aspect of phthisis, the authors averring that "by tuberculosis Nature weeds out the unfit" is a "grossly inaccurate" statement. But is it? Is not the boot rather upon the other leg? Tuberculosis is about thirty times commoner among lunatics and idiots than among the sane, spite of an open-air life in a modern asylum. Such an authority as Sir Frederick Mott accounts for this by their low vitality, and expressly attributes to the disease a racially protective action. Again, any text-book mentions Beneke's work on the congenitally small vascular and alimentary system of consumptives, the former confirmed by modern radiology; as also the frequent ending of congenital heart disease by consumption. Sir Clifford Allbutt, who, as he showed the other day, can still give us admirable surveys of medicine, contributes an interesting and benevolent preface. The book is well printed, of course, and there is a good index, compiled by Mr. F. G. Binnie. It is a sad subject, this, of the fate of the working-class consumptive, sad for the reason that only a vast political and economic change can do much in the way of betterment—a remedy, therefore, that will take time to apply. The object-lesson of all such efforts as the one here described is, however, all to the good. In several places the authors show themselves alive to the fact that the upper middle-class man will often recover where the lower-class patient is doomed. That fact is the key to the whole situation.

**First Aid to the Injured and Sick: an Advanced Ambulance Handbook.** By Drs. Warwick and Tunstall. Eleventh Edition. (Bristol: Wright & Sons.) 1920. Pp. 246. Price 2s. 6d. (paper); 5s. (leather).

This old-established favourite emerges from the Great War triumphantly and recognisably itself. Indeed, at a brief glance the main novelty consists in the expansion of the subject of poisoning by gases. No doubt there is plenty of war experience incorporated in other chapters as well; but it is a fine testimonial to the excellence of the original design and construction that no really radical alteration has proved to be necessary. The authors must by this time have grown grey in the cause of ambulance education; if the Order of St. John of Jerusalem were really alive to its responsibilities, we suggest that they might well have received before now more recognition of their labour than seems to have been vouchsafed them.

**A Text-book of Pharmacology and Medical Treatment for Nurses.** By J. M. Fortescue-Brickdale, M.D., M.R.C.P. Price 25s. net. (H. Frowde and Hodder & Stoughton, Oxford Medical Publications, 17 Warwick Square, London, E.C. 4.)

The author of a book when he sets out intelligibly to explain to nurses the true principles of the medical treatment which they administer on behalf of the medical man is attempting a peculiarly difficult task. It is so easy to be over technical, to elaborate and explain just a little too far, and yet at the same time modern methods and training require of the nurse so much more than mere rule of thumb teaching. We feel, therefore, particularly inclined to congratulate the author of this work upon the skill with which he has steered a happy middle course and provided the nursing profession at once with a reference volume and a valuable text-book. It is divided into two parts, the first being devoted to pharmacology, and a clear, if necessarily restricted, outline is given of the physiological effects of the various groups of drugs, in order that their possibilities and limitations as curative agents may be appreciated. The second section deals with the practical treatment of disease. Throughout the author has used the accepted medical teachings of the best-known authorities of the day, and we can confidently recommend his collection as a reliable guide to those fields which are covered.

**Why Do We Die?** By T. B. Scott, M.R.C.S. (Fisher Unwin. 1921. Pp. 123. 6s net.)

According to the author arteriosclerosis is the main reason—apart from "accidents" such as pneumonia, aeroplane smashes, cancer, and the like—why we do not live for ever, or at least for a great many more years than is usual. He is also convinced that he knows both the preventive and the curative treatment of arteriosclerosis; and he sets out to explain them to non-medical readers. Much of his gospel of hygiene is sound enough, but he makes too many unsupported assertions and wanders much too far into the realms of conjectural hypothesis to be taken seriously from a scientific point of view.

**A Complete Handbook of Midwifery.** By J. K. Watson, M.D.Edin. (London: The Scientific Press, Ltd., 28 and 29 Southampton Street, Strand, W.C. 2. Price 11s.)

The writings of this author upon the subject of practical nursing in its various branches are too well known to need comment or introduction. The present and fourth edition of "A Complete Handbook of Midwifery" has undergone a careful revision, but differs immaterially from its predecessors. Throughout the text is simple and explicit; the information essentially practical, but elementary, more particularly fitted for cottage or isolated midwife. Certain nursing points in the after-treatment of a normal labour show the writer to be of the old school of obstetrics, and modern teachers would deprecate a midwife in any circumstances of British midwifery taking the responsibility of suturing the perineum. A short chapter is given to the causation and prevention of septic post-partum fevers. The book is usefully and generously illustrated, having also an appendix on gynæcological work and a full set of Rules of the Central Midwives Board.

